# Entropy optimized dissipative flow of hybrid nanofluid in the presence of non-linear thermal radiation and Joule heating

**DOI:** 10.1038/s41598-021-95604-4

**Published:** 2021-08-09

**Authors:** Wei-Feng Xia, M. U. Hafeez, M. Ijaz Khan, Nehad Ali Shah, Jae Dong Chung

**Affiliations:** 1 School of Engineering, Huzhou University, Huzhou, 313000 People’s Republic of China; 2 Department of Mathematics, Quaid-I-Azam University 45320, Islamabad, 44000 Pakistan; 3 Department of Mathematics and Statistics, Riphah International University I-14, Islamabad, 44000 Pakistan; 4 Nonlinear Analysis and Applied Mathematics (NAAM)-Research Group, Department of Mathematics, Faculty of Sciences, King Abdulaziz University, P.O. Box 80203, Jeddah, 21589 Saudi Arabia; 5 Department of Mechanical Engineering, Sejong University, Seoul, 05006 Korea; 6 Department of Mathematics, Lahore Leads University, Lahore, Pakistan

**Keywords:** Engineering, Mathematics and computing

## Abstract

Present article reads three dimensional flow analysis of incompressible viscous hybrid nanofluid in a rotating frame. Ethylene glycol is used as a base liquid while nanoparticles are of copper and silver. Fluid is bounded between two parallel surfaces in which the lower surface stretches linearly. Fluid is conducting hence uniform magnetic field is applied. Effects of non-linear thermal radiation, Joule heating and viscous dissipation are entertained. Interesting quantities namely surface drag force and Nusselt number are discussed. Rate of entropy generation is examined. Bvp4c numerical scheme is used for the solution of transformed O.D.Es. Results regarding various flow parameters are obtained via bvp4c technique in MATLAB Software version 2019, and displayed through different plots. Our obtained results presents that velocity field decreases with respect to higher values of magnetic parameter, Reynolds number and rotation parameter. It is also observed that the temperature field boots subject to radiation parameter. Results are compared with Ishak et al. (Nonlinear Anal R World Appl 10:2909–2913, 2009) and found very good agreement with them. This agreement shows that the results are 99.99% match with each other.

## Introduction

Boundary layer flow over a stretched surface has a key importance in both experimental and theoretical point of views. When surface stretches with certain velocity, it develops an in viscid flow immediately, but the viscous flow near the sheet improves slowly, and it takes a certain instant of time to become a fully developed steady flow. Hayat et al.^1^ studied the flow of Maxwell fluid over a stretching surface. Andersson et al.^2^ examined the viscoelastic and electrically conducting flow over a stretching sheet. Kabeir et al.^3^ discussed the mechanism of heat and mass transfer of power law fluid past a stretching sheet in the presence of chemical reaction and radiation effects.

Fastest mode of thermal transport is radiation in which heat transfers in the form of electromagnetic waves without any dependency of medium. Hayat et al.^4^ analyzed the effects of non-linear thermal radiation on the entropy optimized flow. Shehzad et al.^5^ addressed the thermal transport mechanism of Jeffrey nanofluid flow in the presence of non-linear thermal radiation. Waqas et al.^6^ investigated the flow on slandering stretching surface by encountering the effects of thermophoresis, Brownain diffusion and non-linear radiation. Kumar et al.^7^ studied the flow of nanofluid over a stretched surface with non-linear radiation and chemical reaction.

Presence of shear forces reasons the work done by the fluid on its adjacent layers and in irreversible processes this work done transfers into heat. This whole thermodynamic process is termed as viscous dissipation. Gebhart et al.^8^ analyzed the dissipative effects in natural convection. Koo et al.^9^ explored the impact of viscous dissipation in micro channels and tubes. Flow of magneto-nanofluid in the presence of viscous dissipation is carried out by Hayat et al.^10^. Mustafa et al.^11^ presented the study of Jeffrey fluid near the stagnation point by considering the dissipative effects.

A thermodynamic term highly associated with irreversible processes is called entropy. This term is deducted from second law of thermodynamics. Entropy calculates the rate disorder and randomness of the system. Bejan et al.^12^ investigated the role of entropy in thermal transport mechanism. Rashidi et al.^13^ presented entropy optimized flow of electrically conducting nanofluid. Hayat et al.^14^ explained entropy impact on flow containing copper and silver nanoparticles.

Heat transfer fluids have very important applications at industrial sides. Since base liquid are bad conductors of heat due to their weak thermal properties hence the heat transfer devices were less efficient. Here nanotechnology played a key role; Choi^15^ was the first to utilize the term nanofluid. He prepared it by inserting nanoparticles in ordinary liquid and he proved the enhancement in thermal transport process. After that, many of the researchers adopted that technique and many experimental and theoretical work were done in this regard. Prasher et al.^16^ presented the brief study of thermal and viscous properties of nanofluid. Sheikholeslami et al.^17^ discussed MFD viscosity effects of mixed convective magneto-nanofluid. New classification of nanotechnology is hybrid nanofluid with enhanced thermal properties. This nanomaterial is consists of two or more than two nanoparticles in ordinary liquid and the obtained results are more powerful than that of nanofluid. Khan et al.^18^ explored the MHD containing rotating flow of hybrid nanofluid with entropy generation. Chamkha et al.^19^ presented the study of hybrid nanofluid in the presence of radiation and Joule heating. Hayat et al.^20^ studied heat transfer enhancement in the flow of hybrid nanofluid.

Our main target in this research work is to examine the transport characteristics of three different types of hybrid nanoparticles i.e., Ethylene Glycol, Copper and Silver in magnetohydrodynamic flow of viscous fluid between two parallel moving surfaces. The considered fluid is electrical conducting subject to applied magnetic field and bounded between two parallel surfaces in which lower surface linearly stretches. Whole system obeys uniform rotation along specified direction. Energy equation includes conduction, non-linear radiation, Ohmic heating and viscous dissipation. According to author observation, no such attempt is yet done on such topic in literature. Entropy rate is calculated. Graphical analysis of surface drag force and Nusselt number are addressed. Transformations are used to convert the non-linear PDEs to ODEs. Bvp4c Numerical approach is used for the solution of transformed system. Table 1 shows the thermo-physical values of base liquid and nanoparticles. Table 2 presents the comparative result of present work with Ishak et al.^21^.

**Table 1:** Transport characteristics of base fluid and nanoparticles^22,23^.

Nanoparticles/base fluid	$k(W/mK)$	$\rho (kg/{m}^{3})$	$\sigma (s/m)$	${c}_{p}(J/kgM)$
Silver (Ag)	429	10,500	$6.30*{10}^{7}$	235
Copper (Cu)	401	8933	$5.96*{10}^{7}$	385
Ethylene glycol (EG)	0.253	1115	$1.10*{10}^{-4}$	2430

**Table 2:** Comparative analysis of Nusselt number for different values of Prandtl number when remaining parameters of temperature equation is zero.

Pr	Ishak et al.^21^	Present work
0.72	0.809	0.806
1.0	1.000	1.000
3.0	1.924	1.923
10.0	3.721	3.720

Some latest literature on fluid flow behavior towards a different geometries is listed in Refs.^24–30^.

## Problem statement

**Figure 1:**
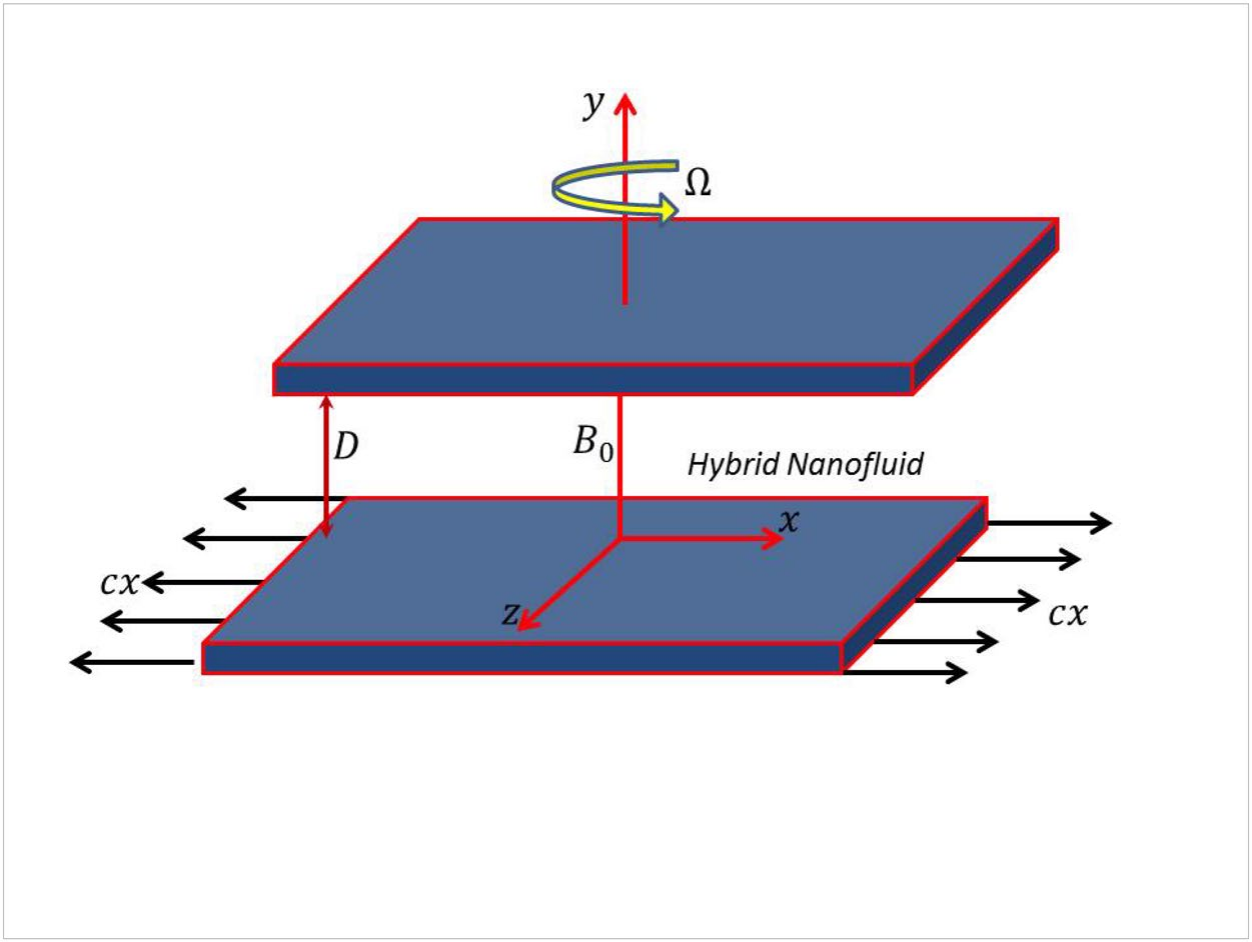
Graphical abstract.

Here we are considering incompressible, steady and viscous flow of hybrid nanofluid bounded between two parallel surfaces which are $D$ distant apart. In hybrid nanomixture, Ethylene glycol $(EG)$ act as a base liquid while copper $(Cu)$ and silver $(Ag)$ as nanoparticles. Since fluid is electromagnetically conducting, hence constant magnetic field $B_{0}$ is applied along $y$ direction by ignoring the electric field effects. There is linear stretching surface at $y = 0$ with stretching velocity $cx$. The considered system is rotating with constant angular velocity $\Omega$ along $y$ direction. Figure 1 shows the physical appearance of the problem.

Mathematical form of the modeled problem is^23^:

1 $$ \frac{\partial u}{{\partial x}} + \frac{\partial v}{{\partial y}} = 0,\, $$

2 $$ u\frac{\partial u}{{\partial x}} + v\frac{\partial u}{{\partial y}} + 2\Omega w = - \frac{1}{{\rho_{hnf} }}\frac{\partial p}{{\partial x}} + \frac{{\mu_{hnf} }}{{\rho_{hnf} }}\left( {\frac{{\partial^{2} u}}{{\partial x^{2} }} + \frac{{\partial^{2} u}}{{\partial y^{2} }}} \right) - \frac{{\sigma_{hnf} B_{0}^{2} }}{{\rho_{hnf} }}u,\, $$

3 $$ u\frac{\partial v}{{\partial x}} + v\frac{\partial v}{{\partial y}} = - \frac{1}{{\rho_{hnf} }}\frac{\partial p}{{\partial y}} + \frac{{\mu_{hnf} }}{{\rho_{hnf} }}\left( {\frac{{\partial^{2} v}}{{\partial x^{2} }} + \frac{{\partial^{2} v}}{{\partial y^{2} }}} \right),\, $$

4 $$ u\frac{\partial w}{{\partial x}} + v\frac{\partial w}{{\partial y}} - 2\Omega u = \frac{{\mu_{hnf} }}{{\rho_{hnf} }}\left( {\frac{{\partial^{2} w}}{{\partial x^{2} }} + \frac{{\partial^{2} w}}{{\partial y^{2} }}} \right) - \frac{{\sigma_{hnf} B_{0}^{2} }}{{\rho_{hnf} }}w,\, $$

5 $$ \left. {\begin{array}{*{20}c} {u\tfrac{\partial T}{{\partial x}} + v\tfrac{\partial T}{{\partial y}} = \tfrac{{k_{hnf} }}{{(\rho c_{p} )_{hnf} }}\left( {\tfrac{{\partial^{2} T}}{{\partial x^{2} }} + \tfrac{{\partial^{2} T}}{{\partial y^{2} }}} \right) - \tfrac{1}{{(\rho c_{p} )_{hnf} }}\tfrac{{\partial q_{r} }}{\partial y} + \tfrac{{\sigma_{hnf} B_{0}^{2} }}{{(\rho c_{p} )_{hnf} }}(u^{2} + w^{2} )} \\ { + \tfrac{{\mu_{hnf} }}{{(\rho c_{p} )_{hnf} }}\left( {2\left( {\tfrac{\partial u}{{\partial x}}} \right)^{2} + 2\left( {\tfrac{\partial v}{{\partial y}}} \right)^{2} + \left( {\tfrac{\partial u}{{\partial y}}} \right)^{2} + \left( {\tfrac{\partial w}{{\partial x}}} \right)^{2} + \left( {\tfrac{\partial w}{{\partial y}}} \right)^{2} } \right).} \\ \end{array} } \right\} $$

On the R.H.S of Eq. (5), first term is due to conduction, second term is due to radiation, third term is due to Joule heating and last term represents the viscous dissipation. By Rosseland's approximation, the non-linear radiative heat flux $q_{r}$ is given as,

6 $$ q_{r} = - \frac{{16\sigma^{ * } T^{3} }}{{3k^{ * } }}\frac{\partial T}{{\partial y}}. $$

The boundary conditions for the present flow satisfy

7 $$ \left. {\begin{array}{*{20}c} {u = cx,\, \, v = 0,\, \, w = 0,\, \, T = T_{0} {\text{ at }}y = 0,} \\ {u = 0,\, \, v = 0,\, \, w = 0,\,T = T_{L} {\text{ at }}y = D.} \\ \end{array} } \right\}\, $$

Here $x,\,y$ highlights Cartesian coordinates, $u,\,v,\,w$ the velocity components, $c$ the stretching rate, $p$ pressure, $\rho_{hnf}$ density, $T$ temperature, $\sigma_{hnf}$ electrical conductivity, $\sigma^{ * }$ Stefan Boltzmann constant, $\mu_{hnf}$ dynamic viscosity, $k^{ * }$ mean absorption coefficient, $\Omega$ angular frequency, $\left( {\rho c_{p} } \right)_{hnf}$ heat capacity, $k_{hnf}$ thermal conductivity. Due to net crossflow along $z - axis,$ $\tfrac{\partial p}{{\partial z}}$ is absent in Eq. (4). The subscript $hnf$ represents hybrid nanofluid.

Thermo-physical aspects of hybrid nanofluid

Hybrid nanofluid dynamic viscosity is given by

8 $$ \frac{{\mu_{hnf} }}{{\mu_{f} }} = \frac{1}{{\left( {1 - \phi_{Cu} - \phi_{Ag} } \right)^{2.5} }}. $$

Density of hybrid nanofluid obeys

9 $$ \frac{{\rho_{hnf} }}{{\rho_{f} }} = \left( {1 - \phi_{Cu} - \phi_{Ag} } \right) + \frac{{\phi_{Cu} \rho_{Cu} + \phi_{Ag} \rho_{Ag} }}{{\rho_{f} }}. $$

Heat capacity of hybrid nanofluid satisfies

10 $$ \frac{{\left( {\rho c_{p} } \right)_{hnf} }}{{\left( {\rho c_{p} } \right)_{f} }} = \left( {1 - \phi_{Cu} - \phi_{Ag} } \right) + \phi_{Cu} \left( {\rho c_{p} } \right)_{Cu} + \phi_{Ag} \left( {\rho c_{p} } \right)_{Ag} . $$

Thermal conductivity of hybrid nanofluid is

11 $$ \frac{{k_{hnf} }}{{k_{f} }} = \frac{{\left( {\tfrac{{\phi_{Cu} k_{Cu} + \phi_{Ag} k_{Ag} }}{{\phi_{Cu} + \phi_{Au} }} + 2k_{f} + 2\left( {\phi_{Cu} k_{Cu} + \phi_{Ag} k_{Ag} } \right) - 2\left( {\phi_{Cu} + \phi_{Ag} } \right)\,k_{f} } \right)}}{{\left( {\tfrac{{\phi_{Cu} k_{Cu} + \phi_{Ag} k_{Ag} }}{{\phi_{Cu} + \phi_{Ag} }} + 2k_{f} - \left( {\phi_{Cu} k_{Cu} + \phi_{Ag} k_{Ag} } \right) - \left( {\phi_{Cu} + \phi_{Ag} } \right)\,k_{f} } \right)}}. $$

Hybrid nanofluid electrical conductivity yield

12 $$ \frac{{\sigma_{hnf} }}{{\sigma_{f} }} = 1 + \frac{{3(\tfrac{{\phi_{Cu} \sigma_{Cu} + \phi_{Ag} \sigma_{Ag} }}{{\sigma_{f} }} - (\phi_{Cu} + \phi_{Ag} ))}}{{(\tfrac{{\phi_{Cu} \sigma_{Cu} + \phi_{Ag} \sigma_{Ag} }}{{\phi \sigma_{f} }} + 2) - (\tfrac{{\phi_{Cu} \sigma_{Cu} + \phi_{Ag} \sigma_{Ag} }}{{\sigma_{f} }} - (\phi_{Cu} + \phi_{Ag} ))}}. $$

Here we have used equal volume concentration of nanoparticles $(\phi_{Cu} = \phi_{Ag} = 0.5\phi )$.

##  Transformation procedure

Here we are considering the following variables

13 $$ \left. {\begin{array}{*{20}c} {u = cxf^{\prime}(\eta ),\, \, v = - cDf(\eta ),\, \, w = cxg(\eta ),} \\ {\theta = \tfrac{{T - T_{0} }}{{T_{D} \, - T_{0} }},\,\eta = \tfrac{y}{D}.} \\ \end{array} } \right\}\,. $$

Conservation law of mass (Eq. 1) is trivially satisfied and the other flow equations yield

14 $$ f^{iv} + Re\frac{{N_{2} }}{{N_{1} }}(ff^{\prime\prime\prime} - f^{\prime}f^{\prime\prime}) - 2Ro\frac{{N_{2} }}{{N_{1} }}g^{\prime} - Mn\frac{{N_{5} }}{{N_{1} }}f^{\prime\prime} = 0,\, $$

15 $$ g^{\prime\prime} + Re\frac{{N_{2} }}{{N_{1} }}(fg^{\prime} - f^{\prime}g) + 2Ro\frac{{N_{2} }}{{N_{1} }}f^{\prime} - Mn\frac{{N_{5} }}{{N_{1} }}g = 0,\, $$

16 $$ \left. {\begin{array}{*{20}c} {[N_{4} + R(1 + (\theta_{w} - 1)\theta )^{3} ]\theta^{\prime\prime} + N_{3} \Pr {\text{Re}} f\theta^{\prime} + 3R(\theta_{w} - 1)(1 + (\theta_{w} - 1)\theta )^{2} \theta^{\prime 2} } \\ { + N_{1} \Pr [Ec_{D} (4f^{\prime 2} + g^{2} ) + Ec_{x} (f^{\prime \prime 2} + g^{\prime 2} )] + N_{5} MnEc_{x} {\text{PrRe}} (f^{\prime 2} + g^{2} ) = 0,} \\ \end{array} } \right\}\, $$

17 $$ \left. {\begin{array}{*{20}c} {f(0) = 0,\, \, f^{\prime}(0) = 1,\, \, g(0) = 0,\, \, \theta (0) = 1{\text{ at }}\eta = 0,} \\ {f(1) = 0,\, \, f^{\prime}(1) = 0,\, \, g(1) = 0,\, \, \theta (1) = 0{\text{ at }}\eta = 1.} \\ \end{array} } \right\}\, $$

Here $\theta_{w} = \left( {\tfrac{{T_{0} }}{{T_{D} }}} \right)$ temperature ratio parameter ($T_{0} > T_{D}$), $Re = \left( {\tfrac{{cD^{2} \rho_{f} }}{{\mu_{f} }}} \right)$ Reynolds number, $\Pr = \left( {\tfrac{{\mu_{f} c_{{p_{f} }} }}{{k_{f} }}} \right)$ Prandtl number, $Mn = \left( {\tfrac{{\sigma_{f} B_{0}^{2} D^{2} }}{{\mu_{f} }}} \right)$ magnetic parameter, $R = \left( {\tfrac{{16\sigma^{ * } T_{D} }}{{3k^{ * } k_{f} }}} \right)$ radiation parameter, $Ro = \left( {\tfrac{{\Omega D^{2} \rho_{f} }}{{\mu_{f} }}} \right)$ rotation parameter, $Ec_{x} = \left( {\tfrac{{c^{2} x^{2} }}{{c_{{p_{f} }} T_{D} (\theta_{w} - 1)}}} \right)$ and $Ec_{D} = \left( {\tfrac{{c^{2} D^{2} }}{{c_{{p_{f} }} T_{D} (\theta_{w} - 1)}}} \right)$ are the Eckert numbers. $N_{1} ,\,N_{2} ,\,N_{3} ,\,N_{4}$ and $N_{5}$ are mathematically given as

18 $$ N_{1} = \frac{{\mu_{hnf} }}{{\mu_{f} }},\, \, N_{2} = \frac{{\rho_{hnf} }}{{\rho_{f} }},\, \, N_{3} = \frac{{\left( {\rho c_{p} } \right)_{hnf} }}{{\left( {\rho c_{p} } \right)_{f} }},\, \, N_{4} = \frac{{k_{hnf} }}{{k_{f} }}{\text{ and }}N_{5} = \frac{{\sigma_{hnf} }}{{\sigma_{f} }} $$

## Entropy generation

Rate of entropy generation is defined as

19 $$ \left. \begin{gathered} E_{G} = \tfrac{{\mu_{hnf} }}{{T_{D} }}\left[ {2\left( {\tfrac{\partial u}{{\partial x}}} \right)^{2} + 2\left( {\tfrac{\partial v}{{\partial y}}} \right)^{2} + \left( {\tfrac{\partial u}{{\partial y}}} \right)^{2} + \left( {\tfrac{\partial w}{{\partial x}}} \right)^{2} + \left( {\tfrac{\partial w}{{\partial y}}} \right)^{2} } \right] \hfill \\ \quad  \quad + \tfrac{{k_{f} }}{{T_{D}^{2} }}\left[ {\tfrac{{k_{hnf} }}{{k_{f} }} + \tfrac{{16\sigma^{ * } T^{3} }}{{3k_{f} k^{ * } }}} \right]\,\left( {\left( {\tfrac{\partial T}{{\partial x}}} \right)^{2} + \left( {\tfrac{\partial T}{{\partial y}}} \right)^{2} } \right) + \tfrac{{\sigma_{hnf} }}{{T_{D} }}B_{0}^{2} (u^{2} + w^{2} ), \hfill \\ \end{gathered} \right\} $$

after applying the transformations, entropy generation becomes

20 $$ \left. {\begin{array}{*{20}c} {Ng = \tfrac{{E_{G} }}{{E_{Go} }} = N_{1} Ec_{D} \Pr (4f^{\prime 2} + g^{2} ) + N_{1} Ec_{x} \Pr (f^{\prime \prime 2} + g^{\prime 2} ) + } \\ {\left( {N_{4} + R(1 + (\theta_{w} - 1)\theta )^{3} } \right)\,\theta^{\prime 2} + N_{5} Mn\Pr Ec_{x} (f^{\prime 2} + g^{2} ).} \\ \end{array} } \right\}\, $$

where $E_{Go} = \left( {\tfrac{{k_{f} (\theta_{w} - 1)}}{{D^{2} }}} \right)$ is the characteristics entropy generation.

## Physical quantities

### Surface drag force

Expression of surface drag force satisfies

21 $$ C_{f}^{ * } = \frac{{ - 2\tau_{w} }}{{\rho_{hnf} (cx)^{2} }},\, $$

where

22 $$ \tau_{w} = \mu_{hnf} \frac{\partial u}{{\partial y}}|_{y = D} ,\, $$

or scalar form is

23 $$ C_{f} Re_{x} = - 2\frac{{N_{1} }}{{N_{2} }}f^{\prime\prime}(1). $$

### Nusselt number

Mathematically one has

24 $$ Nu^{ * } = \frac{{Dq_{w} }}{{k_{f} (T_{D} - T_{0} )(\theta_{w} - 1)}},\, $$

where

25 $$ q_{w} = - \left[ {k_{hnf} + \frac{{16\sigma^{ * } T^{3} }}{{3k^{ * } }}} \right]\,\frac{\partial T}{{\partial y}}|_{y = D} ,\, $$

The final form is

26 $$ Nu = - \left[ {N_{4} + R\left( {1 + \left( {\theta_{w} - 1} \right)\theta \left( 1 \right)} \right)^{3} } \right]\,\theta^{\prime}(1). $$

## Discussion

Here the dissipative flow of hybrid nanofluid with entropy generation is discussed. Impact of interesting parameters namely magnetic parameter $Mn,$ rotation parameter $Ro,$ Reynolds number $Re,$ temperature ratio parameter $\theta_{w} ,$ radiation parameter $R,$ and Eckert number $Ec_{x}$ are examined.

**Figure 2:**
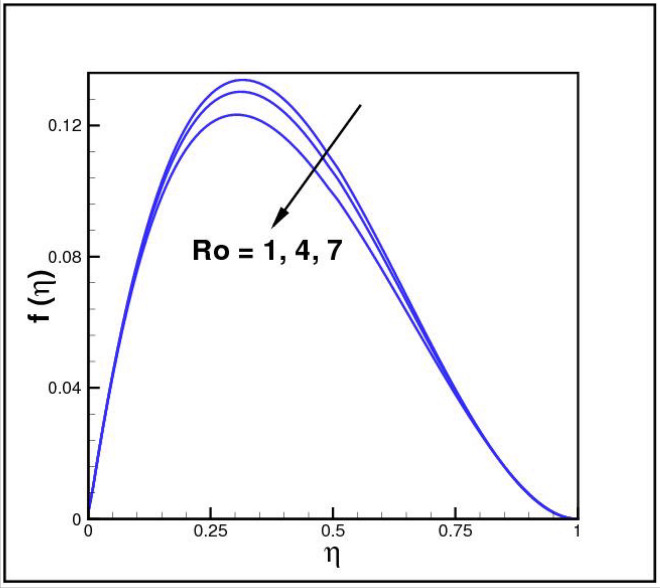
Impact of *Ro* on *f*(*η*).

**Figure 3:**
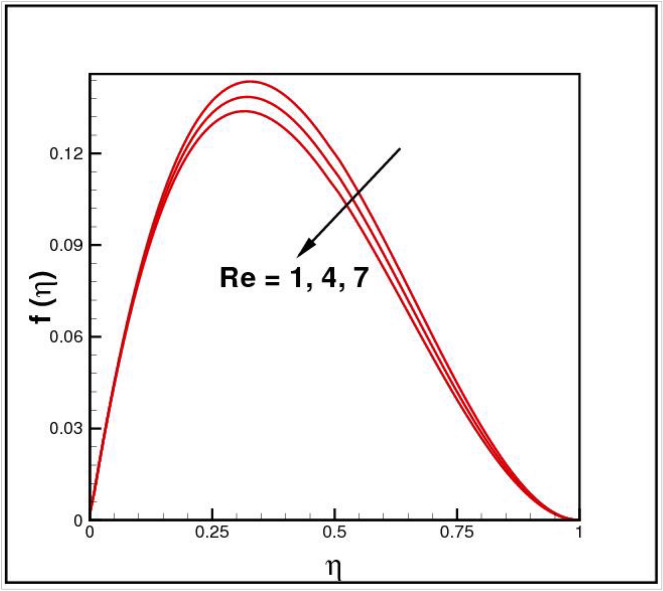
Impact of *Re* on *f*(*η*).

**Figure 4:**
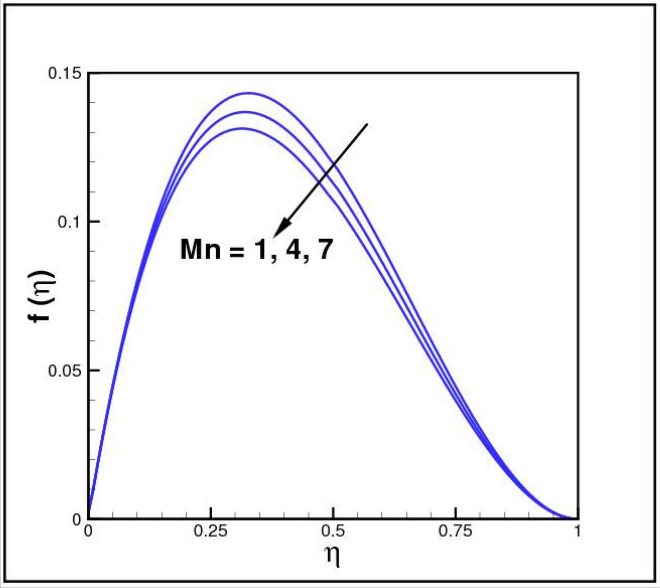
Impact of *Mn* on *f*(*η*).

**Figure 5:**
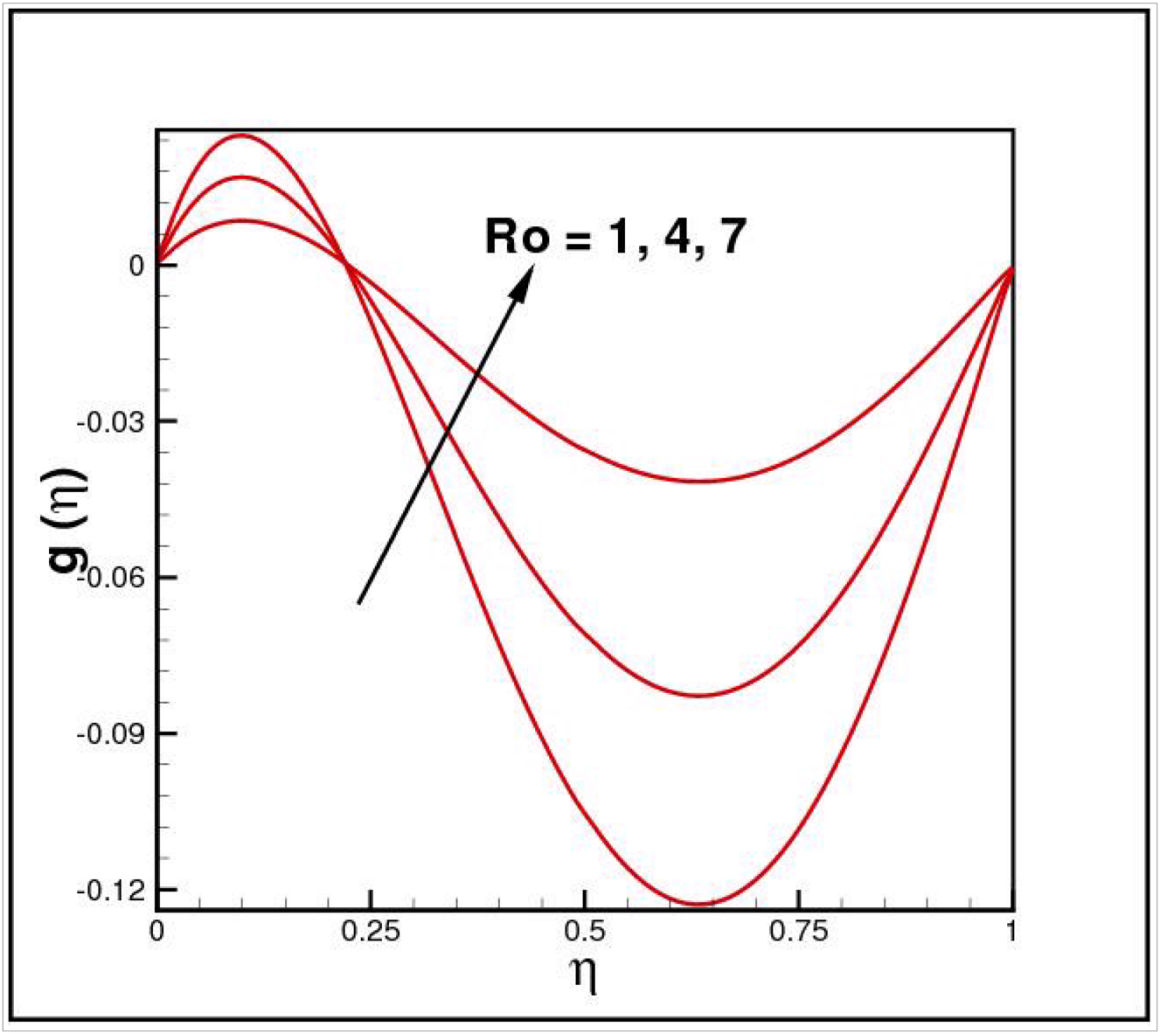
Impact of *Ro* on *g*(*η*).

**Figure 6:**
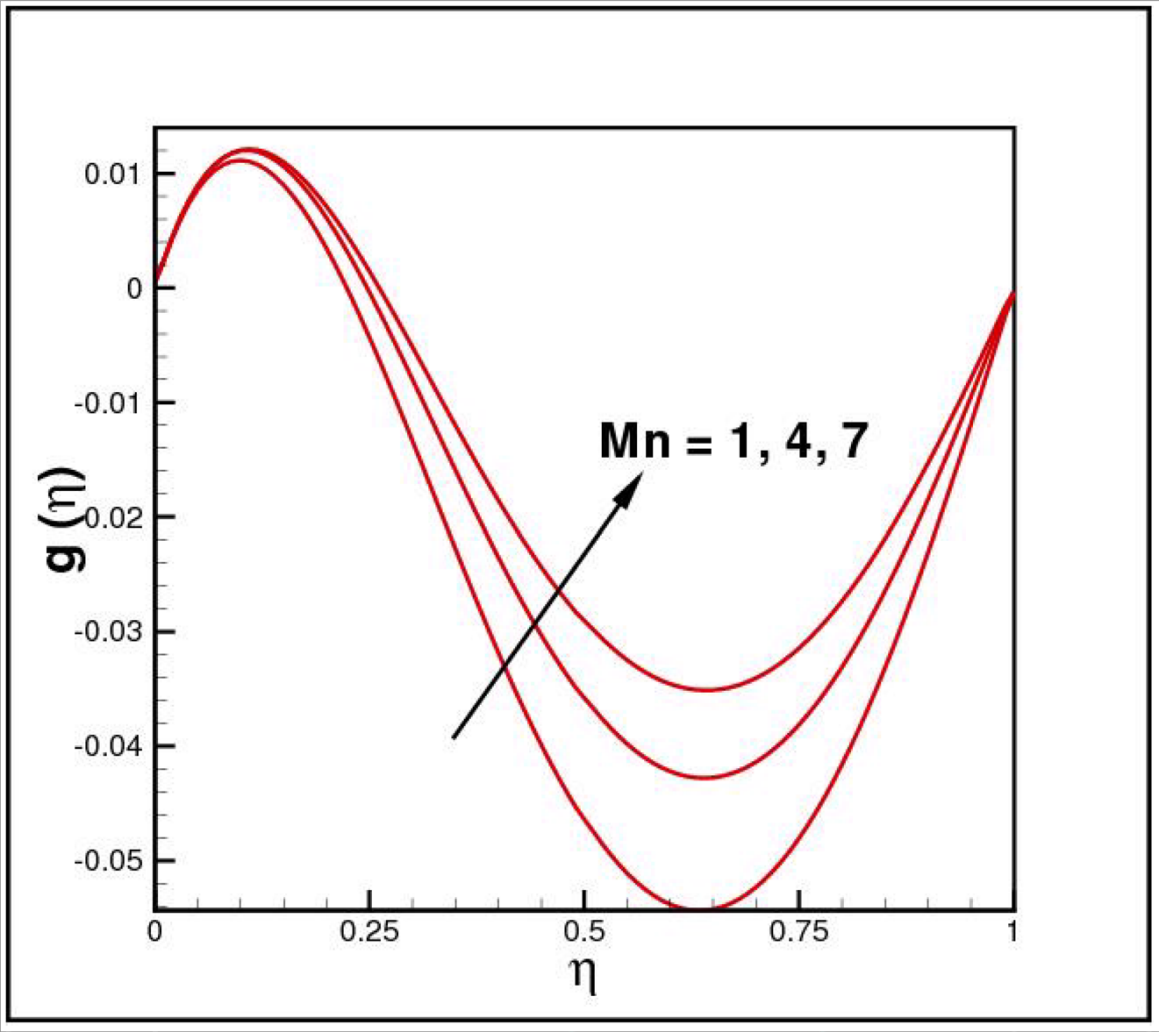
Impact of *Mn* on *g*(*η*).

**Figure 7:**
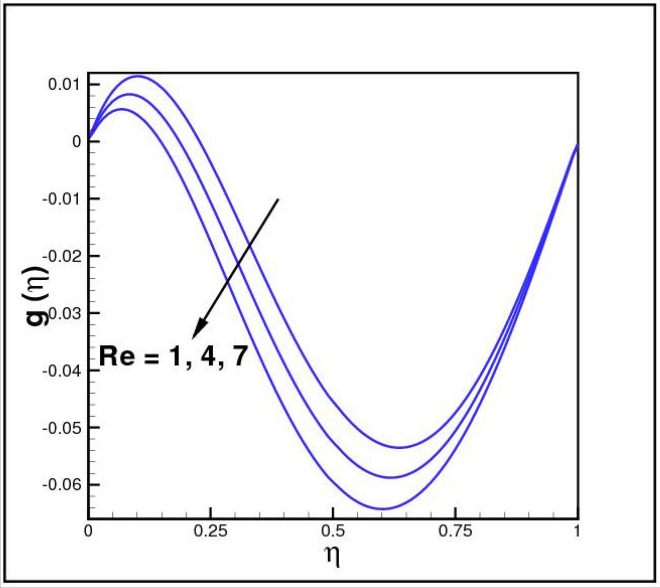
Impact of *Re* on *g*(*η*).

**Figure 8:**
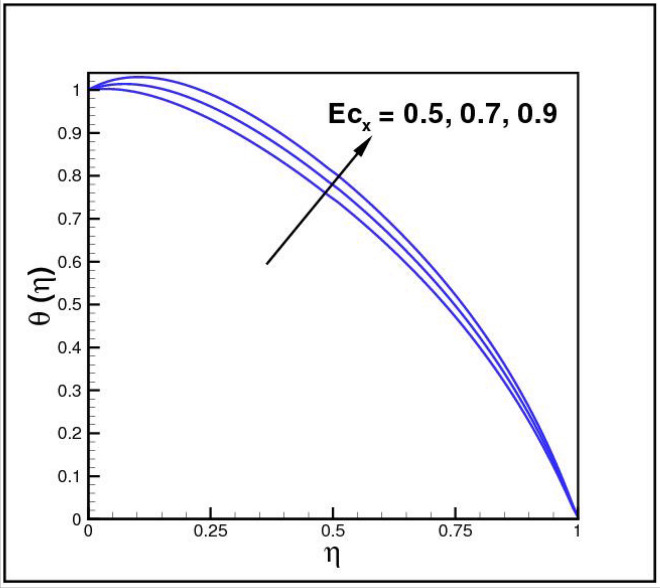
Impact of *Ec*_*x*_ on *θ*(*η*).

**Figure 9:**
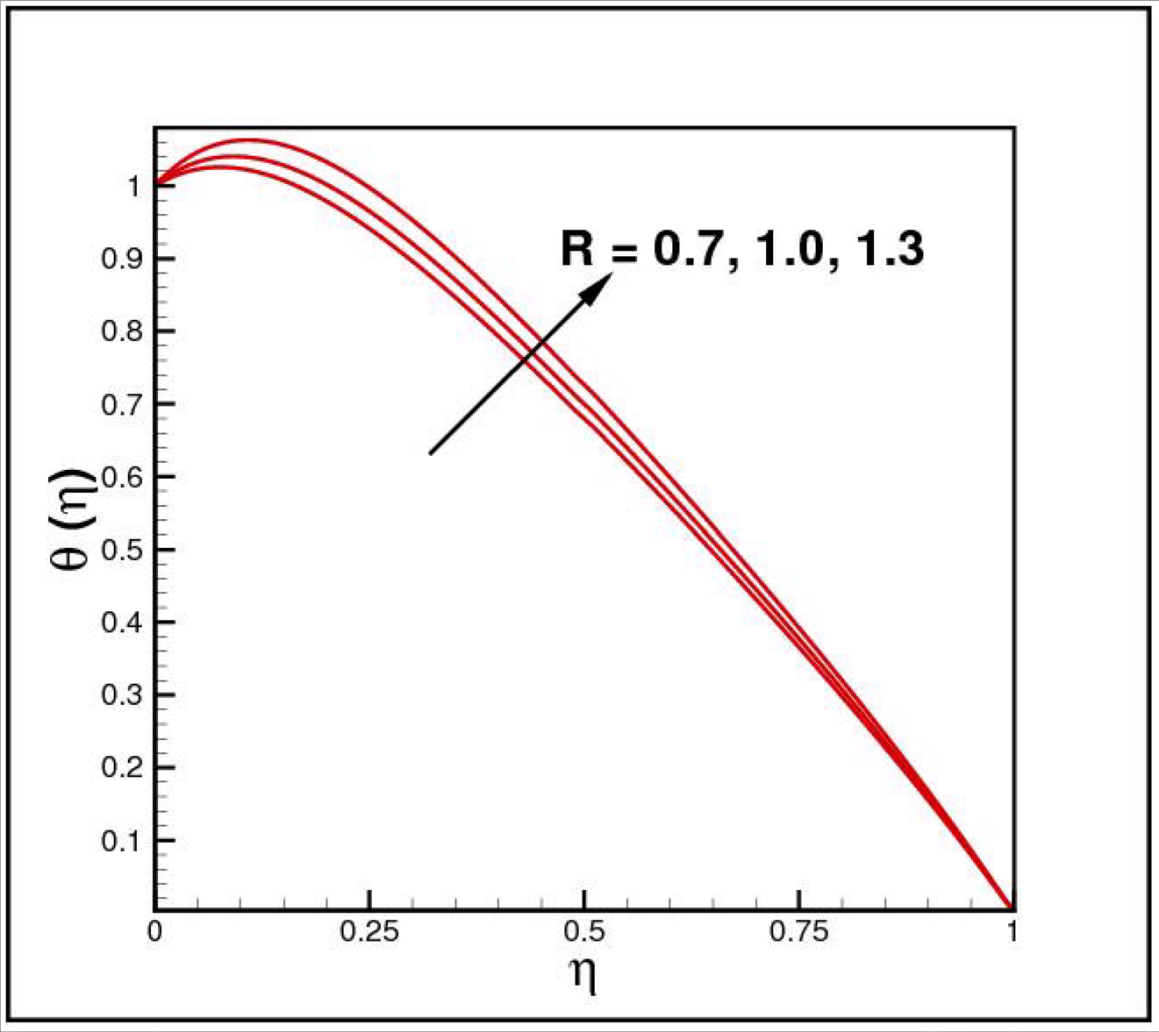
Impact of *R* on *θ*(*η*).

**Figure 10:**
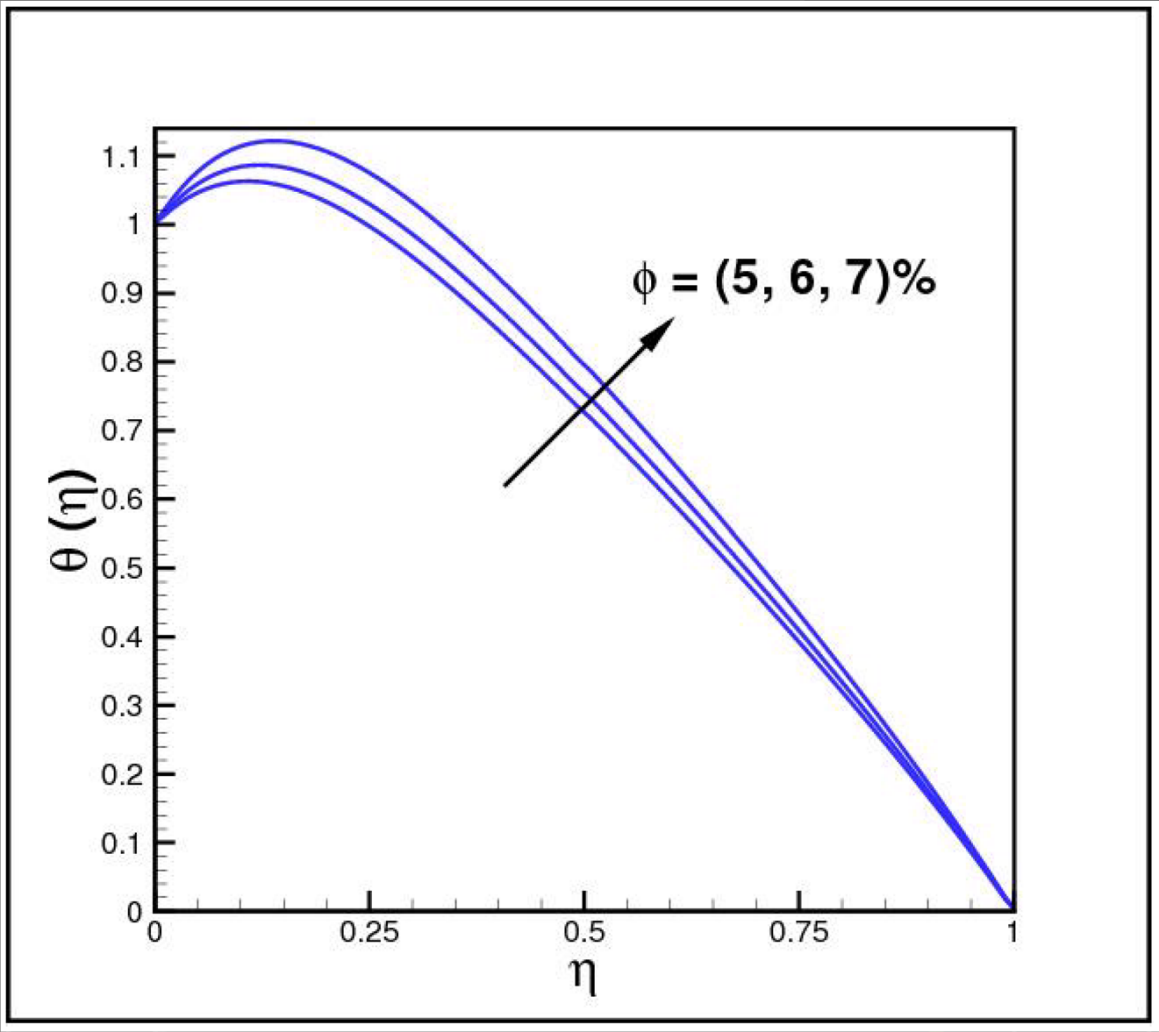
Impact of *ϕ* on *θ*(*η*).

**Figure 11:**
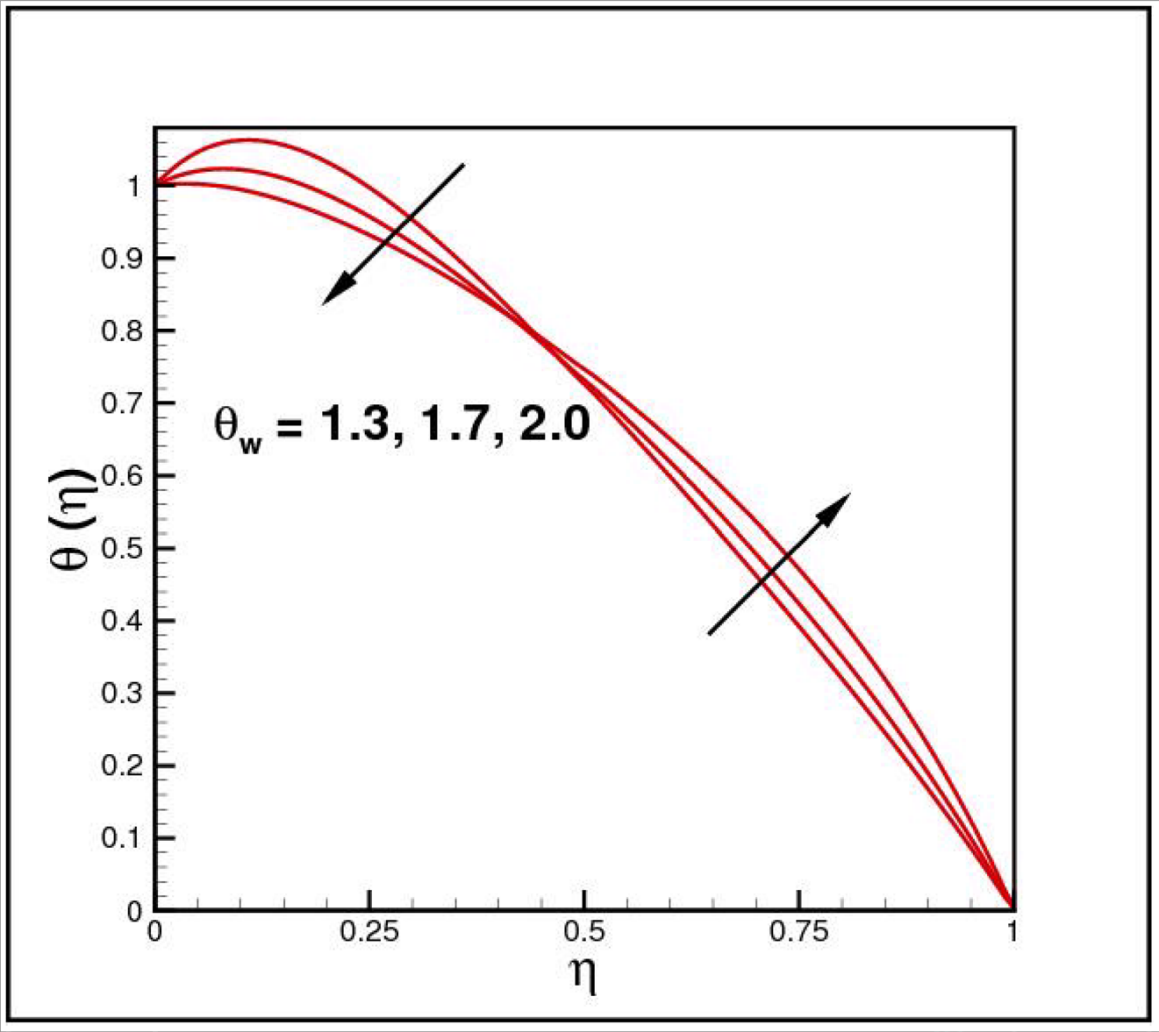
Impact of *θ*_*w*_ on *θ*(*η*).

**Figure 12:**
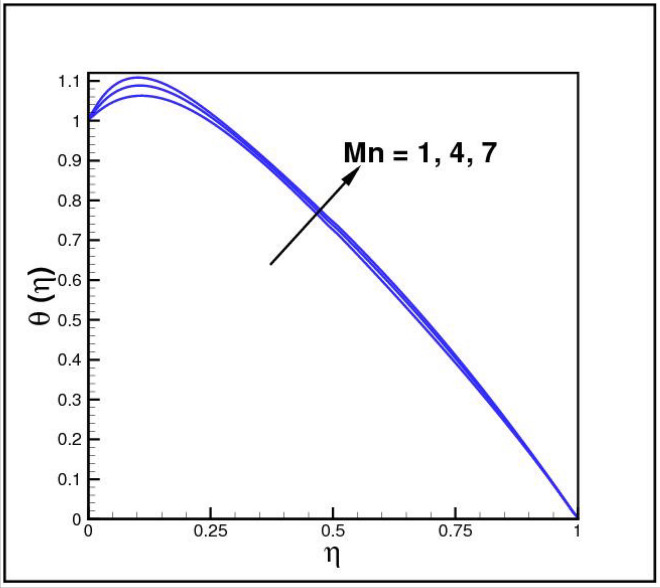
Impact of *Mn* on *θ*(*η*).

**Figure 13:**
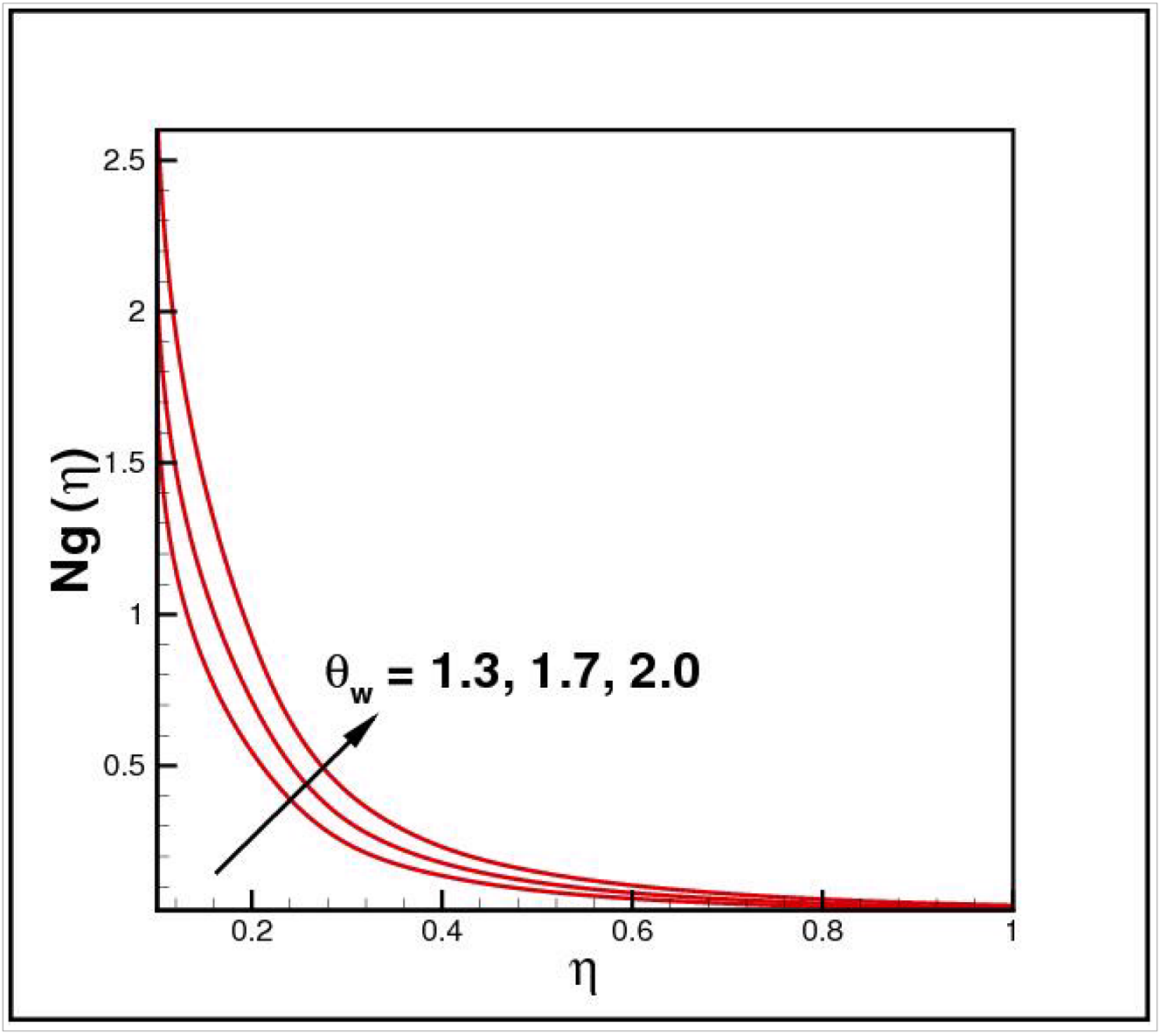
Impact of *θ*_*w*_ on *Ng*(*η*).

**Figure 14:**
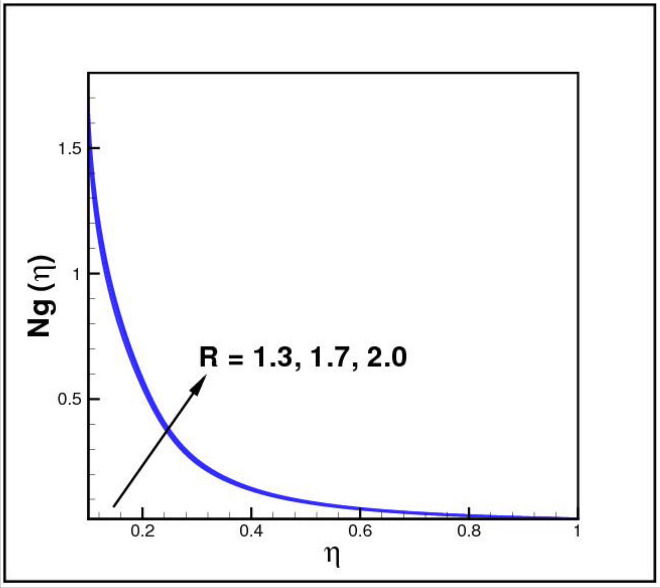
Impact of *R* on *Ng*(*η*).

**Figure 15:**
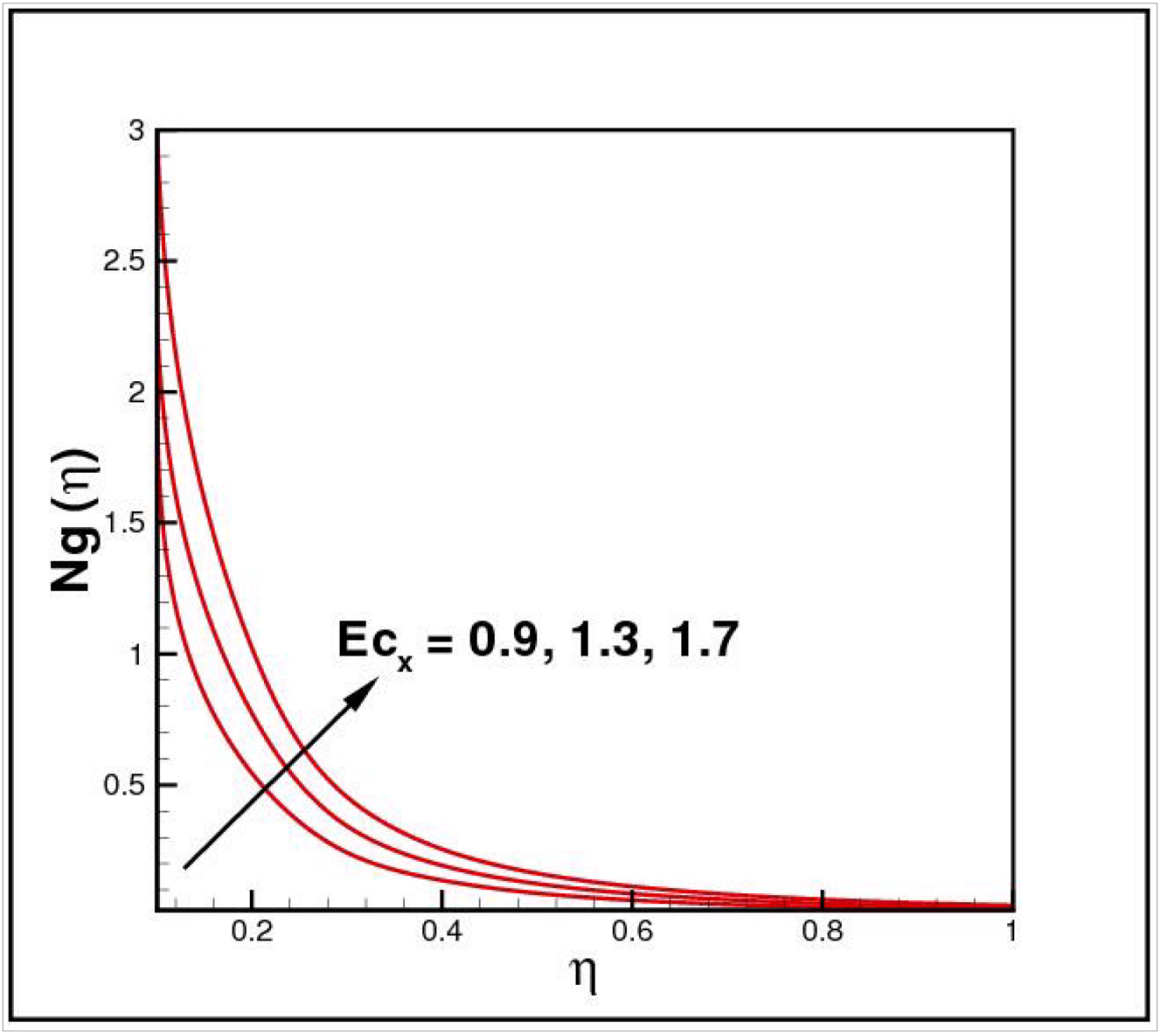
Impact of *Ec*_*x*_ on *Ng*(*η*).

**Figure 16:**
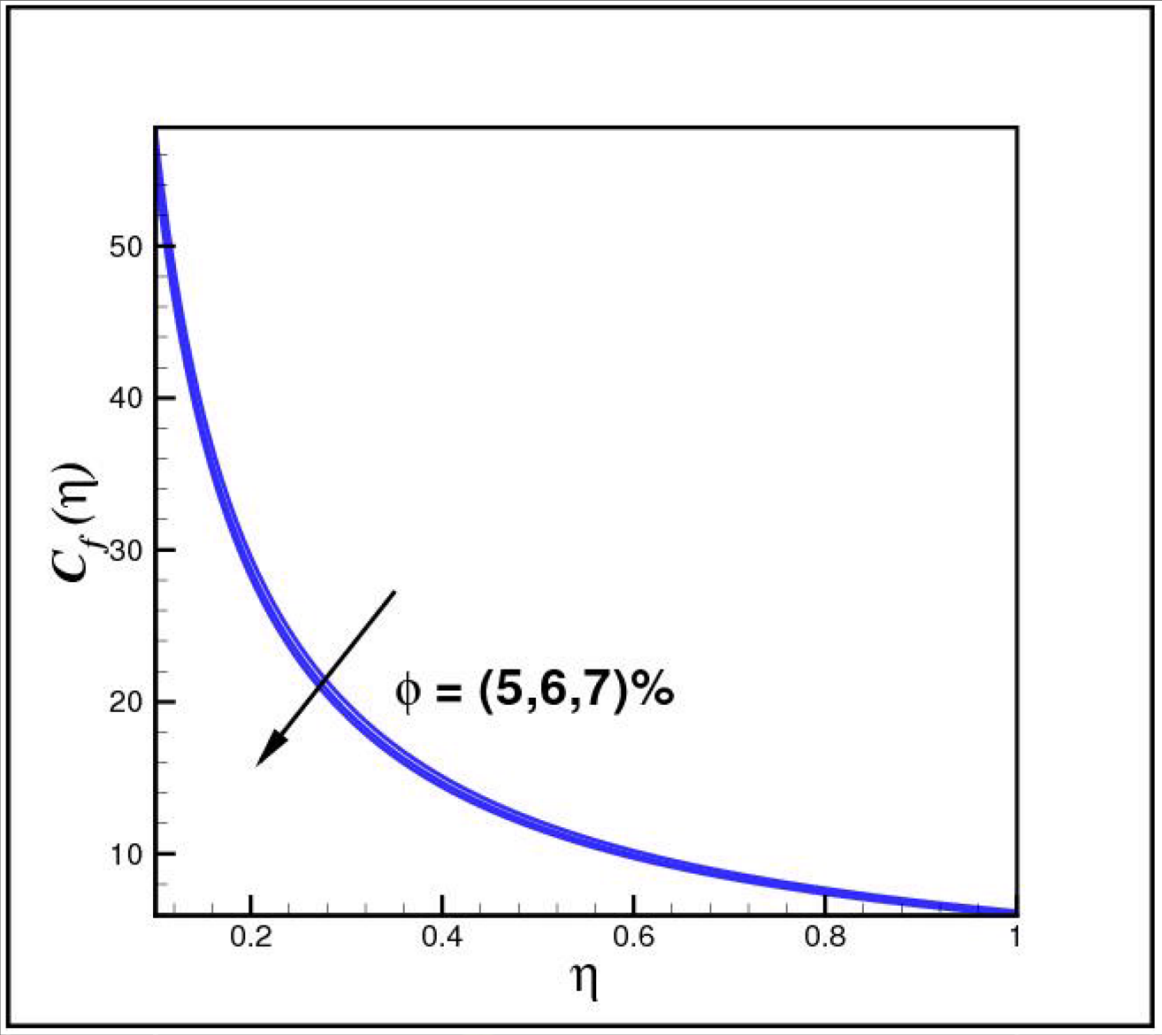
Impact of *ϕ* on *C*_*f*_(*η*).

**Figure 17:**
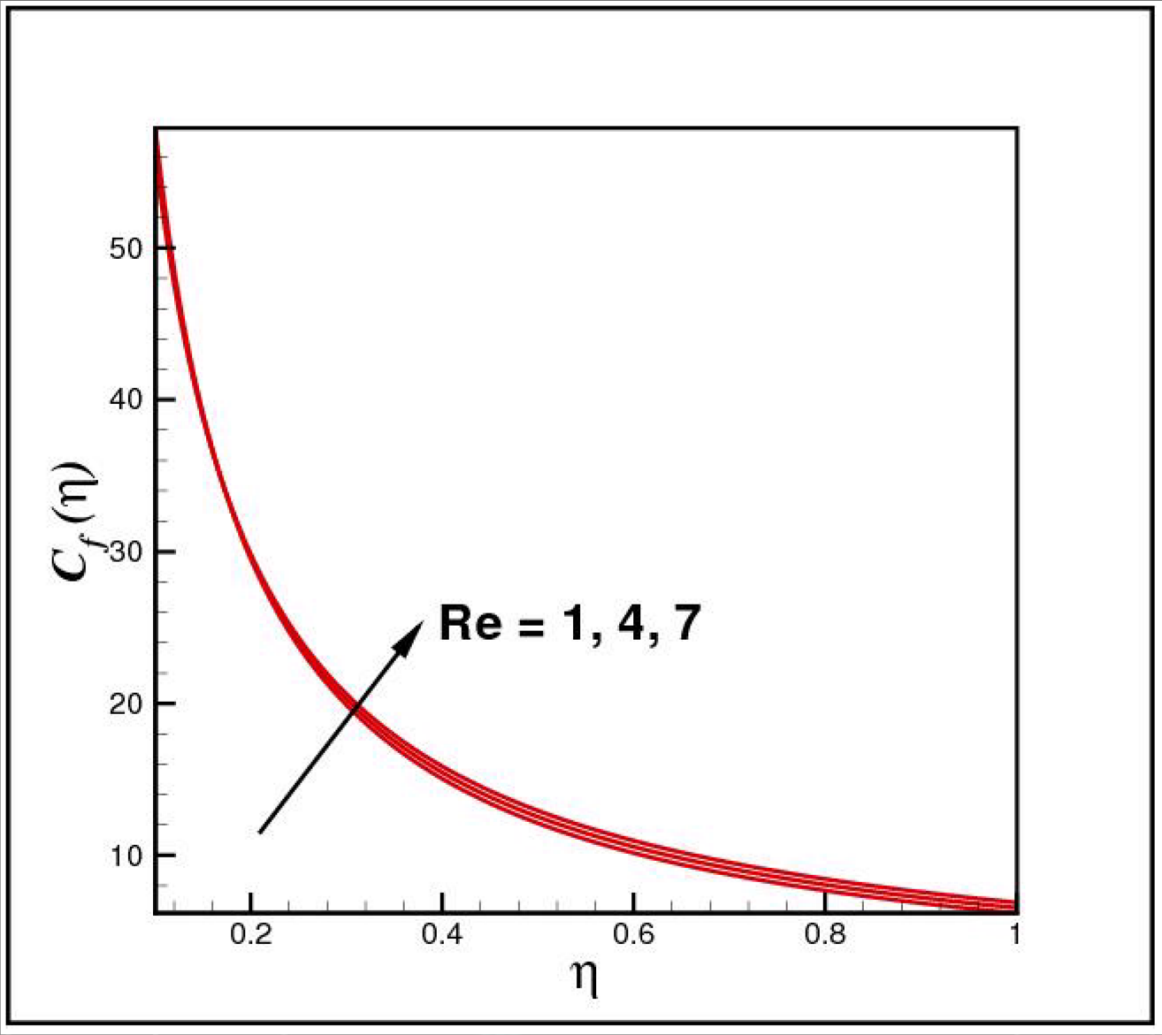
Impact of *Re* on *C*_*f*_(*η*).

**Figure 18:**
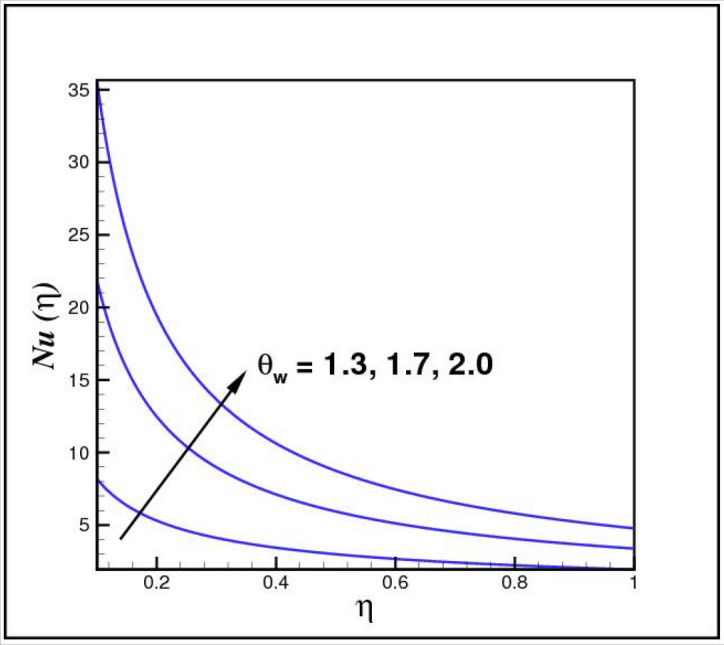
Impact of *θ*_*w*_ on *Nu*(*η*).

**Figure 19:**
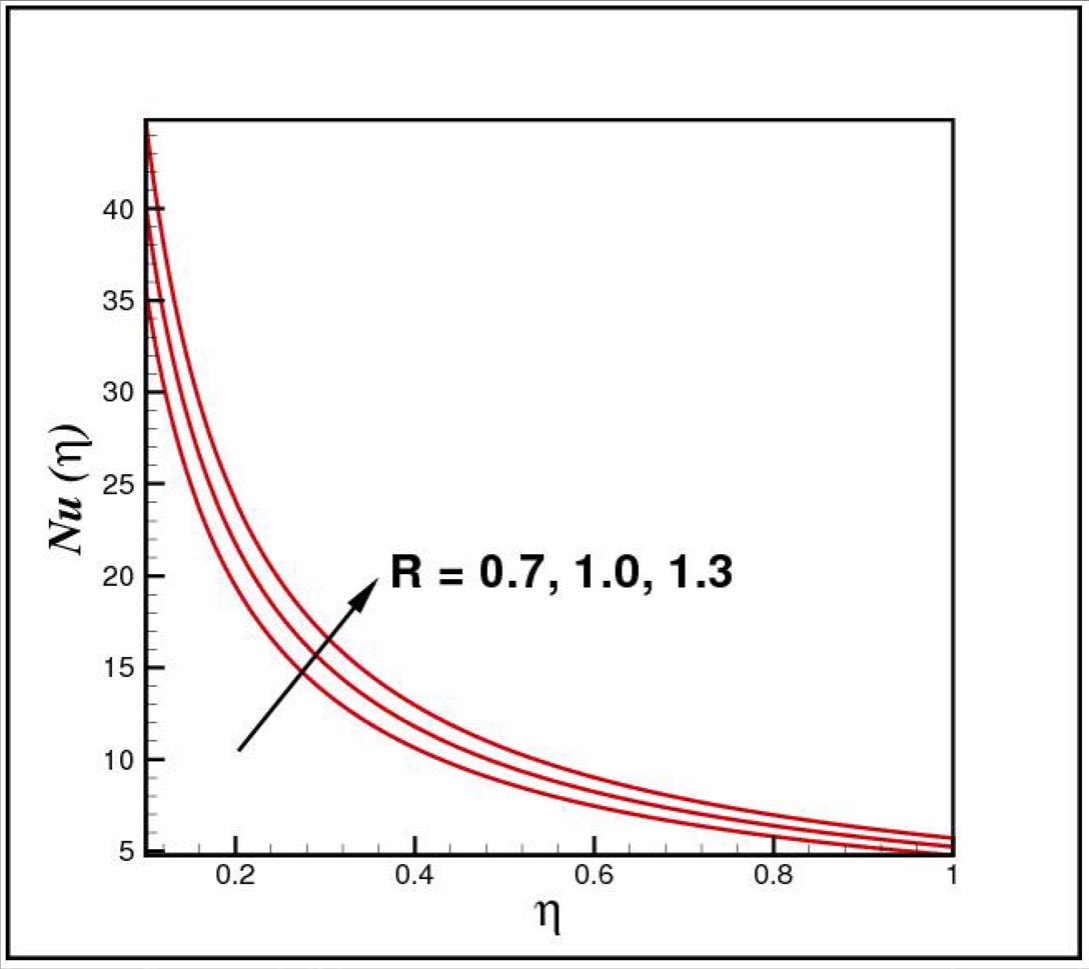
Impact of *R* on *Nu*(*η*).

Figures 2, 3 and 4 present the influences of rotation parameter $Ro$, Reynolds number $Re$ and magnetic parameter $Mn$ on velocity component $f(\eta ),$ respectively. Here $f(\eta )$ is decreasing function of all such parameters. Physically more $Mn$ produces more Lorentz force which offers resistance to flow. Figures 5 and 6 portray the impacts of $Ro$ and $Mn$ on velocity profile $g(\eta ),$ higher values of both parameters reasons the enhancement in $g(\eta )$, while opposite trend is noted for Reynolds number $Re,$ here higher $Re$ declines the velocity $g(\eta )$ as shown in Fig. 7. Figure 8 is plotted to examine the behavior of Eckert number $Ec_{x}$ against temperature $\theta (\eta ),$ since $Ec_{x}$ is a relation between kinetic energy and enthalpy, increase in $Ec_{x}$ causes increase of kinetic energy which further rises up the molecular motion and hence temperature rises. Figure 9 is sketched to see the variation of radiation parameter $R$ on temperature $\theta (\eta )$. It is observed that $\theta (\eta )$ enhanced versus higher $R.$ Figure 10 plots the temperature $\theta (\eta )$ for various percentages of volume fraction of nanoparticles $\phi .$ Clearly $\theta (\eta )$ enhances with an increase in $\phi .$ From Fig. 11 it is observed that for higher estimates of temperature ratio parameter $\theta_{w} ,$ temperature $\theta (\eta )$ inclines near the lower surface while declines near upper boundary. Figure 12 shows the effect of magnetic parameter $Mn$ on temperature $\theta (\eta ),$ since $Mn$ is a resistive body force hence larger $Mn$ causes increment in $\theta (\eta ).$ Figures 13, 14 and 15 exhibit the dimensionless entropy generation $Ng(\eta )$ for different values of temperature ratio parameter $\theta_{w} ,$ radiation parameter $R$ and Eckert number $Ec_{x}$ respectively. An enhancement is observed in $Ng(\eta )$ versus higher values of all parameters. Figure 16 describes the variation in surface drag force $C_{f} (\eta )$ due to volume fraction of nanoparticles $\phi .$ Here higher $\phi$ reasons lower $C_{f} (\eta ).$ Figure 17 demonstrates the impact of Reynolds number $Re$ against $C_{f} (\eta ).$ Clearly $C_{f} (\eta )$ shows increasing behavior for larger $Re.$ Figures 18 and 19 explored effects of temperature ratio parameter $\theta_{w}$ and radiation parameter $R$ on Nusselt number $Nu(\eta ).$ Increment in $Nu(\eta )$ is noticed for the higher values of both parameters.

Table 2 is constructed for the comparative analysis of present work with Ishak et al.^21^ and observed very good agreement with them.

## Concluding remarks

Here the flow analysis of $Ag - Cu/EG$ hybrid nanofluid is discussed. Key findings are listed below.

- Velocity $f(\eta )$ is the decreasing function of higher $Re$ and $Mn$.
- Velocity $g(\eta )$ enhances against higher $Mn$ while it decays against the estimation of $Re.$
- Increment in temperature $\theta (\eta )$ is seen for higher $R$ and $Mn.$
- $C_{f}$ is enhanced for $Re$ while it declined against $\phi .$
- $Ng(\eta )$ rises versus higher $Ec_{x} .$
- Magnitude of $Nu$ is an increasing function of $R$ and $\theta_{w} .$

## Data Availability

The data that support the findings of this study are available within the article, the data are made by the authors themselves and do not involve references of others.
